# Synaptotagmin 4 Supports Spontaneous Axon Sprouting after Spinal Cord Injury

**DOI:** 10.1523/JNEUROSCI.1593-23.2024

**Published:** 2024-09-12

**Authors:** Kyoka Higuchi, Akiko Uyeda, Lili Quan, Shogo Tanabe, Yuki Kato, Yukio Kawahara, Rieko Muramatsu

**Affiliations:** ^1^Department of Molecular Pharmacology, National Institute of Neuroscience, National Center of Neurology and Psychiatry, Kodaira, Tokyo 187-8502, Japan; ^2^Department of NCNP Brain Physiology and Pathology, Graduate School of Medical and Dental Sciences, Tokyo Medical and Dental University, Tokyo 113-8510, Japan; ^3^Department of RNA Biology and Neuroscience, Graduate School of Medicine, Osaka University, Suita, Osaka 565-0871, Japan

**Keywords:** axon regeneration, corticospinal tract, spinal cord

## Abstract

Injuries to the central nervous system (CNS) can cause severe neurological deficits. Axonal regrowth is a fundamental process for the reconstruction of compensatory neuronal networks after injury; however, it is extremely limited in the adult mammalian CNS. In this study, we conducted a loss-of-function genetic screen in cortical neurons, combined with a Web resource-based phenotypic screen, and identified synaptotagmin 4 (Syt4) as a novel regulator of axon elongation. Silencing Syt4 in primary cultured cortical neurons inhibits neurite elongation, with changes in gene expression involved in signaling pathways related to neuronal development. In a spinal cord injury model, inhibition of Syt4 expression in cortical neurons prevented axonal sprouting of the corticospinal tract, as well as neurological recovery after injury. These results provide a novel therapeutic approach to CNS injury by modulating Syt4 function.

## Significance Statement

Promoting axonal regrowth has been considered a promising therapeutic target for functional recovery after central nervous system injury. In the present study, we used a Web resource-based phenotypic screening followed by loss-of-functional screening for neurite elongation. We identified a novel role of synaptotagmin 4 (Syt4), a well-characterized regulator of synaptic function, in neurite elongation. In addition, Syt4 knockdown in the corticospinal tract inhibited axonal regrowth and functional recovery after spinal cord injury. In this way, we provide a new screening method, which allows us to efficiently find new targets regulating CNS therapeutics.

## Introduction

Damage to the central nervous system (CNS) causes severe and persistent neurological dysfunction associated with neuronal network disruption (Raineteau and Schwab, 2001). Although degenerated CNS axons spontaneously regrow through collateral sprouting and form compensatory neuronal networks after injury, they are severely restricted and have limited capacity to restore lost neurological function in adult mammals (Raineteau and Schwab, 2001; Rosenzweig et al., 2010). Thus, elucidating the mechanism that facilitates spontaneous CNS axon regrowth is essential for developing therapies for patients with CNS damage. The mechanisms of CNS axon regrowth are associated with prodegenerative neuronal changes in response to injury (Fink et al., 2017; Poplawski et al., 2020). Because the adult CNS environment contains factors that inhibit axon regrowth, reactive intrinsic growth programs following injury have been interpreted as intracellular signals that mediate axon regrowth (Mahar and Cavalli, 2018; Tran et al., 2018).

Recent advances in screening techniques have provided new opportunities to identify novel target molecules related to neurological diseases. For instance, genome-wide RNAi gene silencing screens have identified novel intrinsic regulators of axon regrowth, such as Sac2 (Zou et al., 2015) and Rab27b (Sekine et al., 2018). Upcoming research should facilitate the identification of new targets with increased translational efficiency for the therapeutics of functional recovery after injury. The development of Web-accessible resources that accumulate knowledge about genotype–phenotype relationships or the gene expression profiling of CNS diseases, both in rodents and humans, would allow us to efficiently prioritize genes for further investigation (Patnala et al., 2013). Nevertheless, the integration of genotype data with pathological phenotypes to identify the key regulator of axon regrowth remains an unexplored challenge.

## Materials and Methods

### Experimental subjects

All animal experiments were approved by the Committee on the Ethics of Animal Experiments of the National Institute of Neuroscience, National Center of Neurology and Psychiatry (2021013-R2). C57BL/6J mice were obtained from Tokyo Laboratory Animal Science and Japan SLC. The mice were housed in groups of three in an air-conditioned room at 23 ± 1°C, with a 12 h light/dark cycle under specific pathogen-free conditions. All mice had *ad libitum* access to water and food. The mice were randomly assigned to groups.

### Primary culture of cortical neurons

Primary cultures of cortical neurons were prepared from the mice on postnatal day 1 (Muramatsu et al., 2012). The cerebral cortices were dissected and dissociated with 0.25% trypsin in phosphate buffer saline (PBS) at 37°C for 15 min. After neutralization with fetal bovine serum (Sigma-Aldrich), the cells were centrifuged at 400 × *g* for 3 min and suspended in 10% fetal bovine serum-Dulbecco's modified Eagle's medium (Thermo Fisher Scientific). Cell suspensions were filtered through a 70 μm nylon cell strainer, and cells were plated on poly-ʟ-lysine (Sigma-Aldrich)-precoated 48-well dishes at a density of 1 × 10^5^ cells/well. Cells were maintained at 37°C and 5% CO_2_.

**Figure 1.**
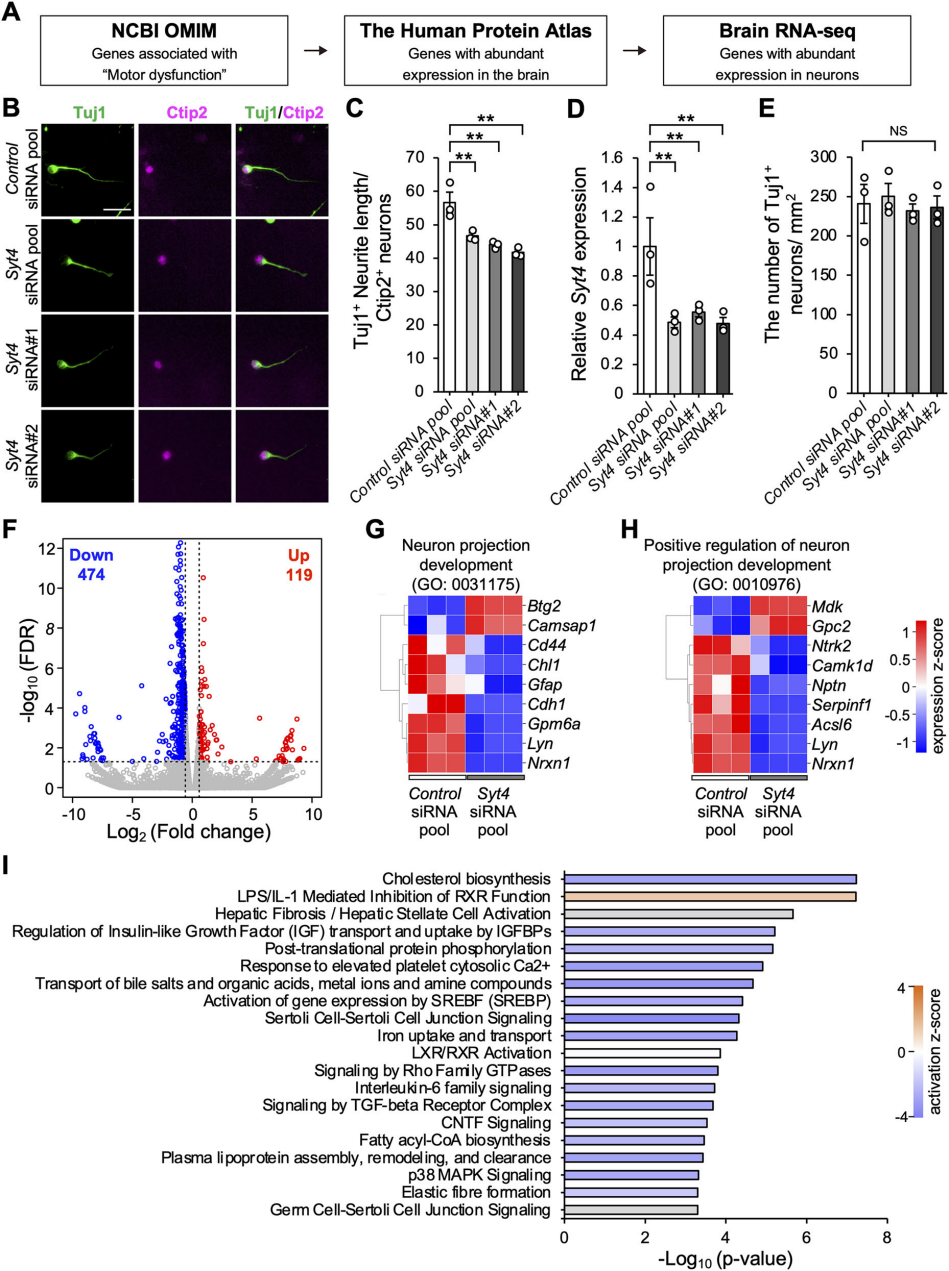
Syt4 promotes neurite elongation in vitro. ***A***, Schematic design for screening to identify the factors involved in neurite elongation from several databases. ***B***, Immunocytochemical images of primary cortical neurons stained with Tuj1 (green) and Ctip2 (magenta) after *Control* siRNA pool, *Syt4* siRNA pool, *Syt4* siRNA#1, or *Syt4* siRNA#2 transfection. Scale bar, 25 µm. ***C***, Quantitative analysis of the average neurite length in cortical neuron cultures (*n *= 3 cultures for each group, **p < 0.01). ***D***, Relative expression of *Syt4* mRNA in the cortical neurons 3 d after indicated siRNA transfection (*n *= 3 cultures for each group, **p < 0.01). ***E***, The number of cortical neurons per mm^2^ after siRNA transfection (*n *= 3 cultures for each group; NS, not significant). ***F***, Volcano plot of RNA-seq data from primary cortical neurons transfected with *Control* or *Syt4* siRNA (*n *= 3 cultures for each group). The red and blue dots represent significantly up- and downregulated genes, respectively. ***G***, ***H***, Heatmap showing the expression level of DEGs annotated with “neuron projection development (GO:0031175, ***G***)” and the “positive regulation of neuron projection development (GO: 0010976, ***H***)” in the gene ontology analysis. ***I***, Graph showing top 20 canonical pathways. Color of bars represent *z*-score of expected activation (positive) or inhibition (negative) state of the pathways calculated using IPA. Gray bars indicate that no activity pattern was identified in IPA, despite significant association of DEGs within the pathway. Data are presented as mean ± standard error of the mean. *p* values were determined using one-way ANOVA with Tukey's post hoc test.

One day after plating, mouse siGENOME siRNAs (Horizon Discovery) for the target genes (Fig. 1*A*, Table 1) were transfected into cultured cortical neurons using Lipofectamine 2000 (Thermo Fisher Scientific). siRNA pools, including four distinct siRNAs targeting different gene regions, were used for the first screening. Additionally, individual siRNAs targeting Synaptotagmin 4 (Syt4; target sequence; siSyt4#1, 5′-GAAATGCACTCCCAACGCA-3′; siSyt4#2, 5′-GAGCTCTCCTTGCACTTTA-3′) were used to validate the on-target effect. To assess the effect of gene silencing on regenerative neurite outgrowth with mimicking in vivo axonal injury, neurons were trypsinized to injure and remove neurites after 3 d of culture. Then the cells were replated and cultured for 1 d to regrow their neurites.

**Table 1.** Neurite outgrowth assay

Target gene	Neurite length (µm)		Relative neurite length to control
	Trial #1	Trial #2	
Control	51	51.6	1
Islr2	53.7	46.5	0.98
Nos1	46.7	47.8	0.92
Magel2	59.7	51.9	1.09
Hcn4	48.3	47.2	0.93
Grm2	53	48.1	0.99
Bhlhe22	39.4	49.9	0.87
Tubb3	48.6	44.3	0.91
Syt4	42.4	43.7	0.84^a^
Calb2	55.6	44.8	0.98
Chrm3	42.5	47.3	0.88
Gabrg2	47	48.6	0.93
Atp1a3	52.4	45.5	0.95
Gad1	49	42.8	0.89
Hcn1	50.8	42.6	0.91
Gria1	52.8	43.4	0.94
Hecw1	46.5	45.5	0.9
Cntnap2	47.2	46.4	0.91
Cacna1a	47.5	46.1	0.91
Slc12a5	54.1	44.7	0.96
Grin1	54.8	41.5	0.94

List of targeted genes in siRNA-based loss-of-function genomic screen in neurite outgrowth. The length of Tuj1-positive neurites and relative length of them to control are indicated.

a Indicates highest inhibition efficiency.

### Immunocytochemistry

Cultured cortical neurons were fixed with 4% paraformaldehyde in PBS for 30 min and permeabilized using 0.1% Triton X-100 in PBS for 10 min at 20–25°C. The samples were treated with blocking buffer (PBS containing 3% normal donkey serum) for 1 h at 20–25°C, followed by incubation with rabbit anti-class III beta-tubulin antibody (Tuj1, 1:2,000, catalog #802001, BioLegend) and rat anti-Bcl11b (Ctip2) antibody (1:500, catalog #650601, BioLegend), diluted in blocking buffer overnight at 4°C. The cells were then treated with the secondary antibody, Alexa Fluor 488 or 594-conjugated donkey antibody against rabbit or rat IgG (Thermo Fisher Scientific), respectively, diluted in blocking buffer for 1 h at 20–25°C. The nuclei were stained with 4′,6-diamidino-2-phenylindole (DAPI, 1 mg/ml, Dojindo Laboratories). Images were acquired using an IN Cell Analyzer 2000 (GE Healthcare) with ps20 × objective lens. The neurite length of >50 Tuj1^+^Ctip2^+^ neurons was measured using ImageJ software, and the average neurite length was calculated for each sample.

### Quantitative reverse transcription-PCR

To evaluate the knockdown efficiency, transfected cells were cultured for additional 3 d and used for real-time PCR analysis. In order to assess the efficacy of overexpression, the brains of mice that received recombinant AAV vectors were collected 2 weeks postsurgery. Using a fluorescent stereomicroscope, the sensory motor cortex regions displaying EYFP expression were carefully excised. Total RNA was isolated using an RNeasy Mini Kit (Qiagen). cDNA was synthesized using a High-Capacity cDNA Reverse Transcriptase Kit (Applied Biosystems). Real-time reverse transcription (RT)-PCR was performed using KAPA SYBR Fast Master Mix (KAPA Biosystems) with the following primer pairs: Syt4 forward, GACAGAGCACGCAGAAAACA; Syt4 reverse, AGTGAAGACGAGGCCAAAAG; Gapdh forward, AGGTCGGTGTGAACGGATTTG; and Gapdh reverse, TGTAGACCATGTAGTTGAGGTCA. PCR conditions included one cycle at 95°C for 30 s, followed by 39 cycles at 95°C for 5 s and 60°C for 45 s. Melting analysis was performed using PCR to monitor amplification specificity. Relative mRNA expression was normalized to Gapdh mRNA levels in the same samples and calculated using the Δ/Δ-Ct method.

### RNA-seq

To evaluate the effect of Syt4 knockdown on mRNA expression in cortical neurons, total RNA was extracted from cortical neuron cultures transfected with Syt4 siRNA using the RNeasy Micro Kit (Qiagen) after replating and on another 1 d of culture. Three biological replicates were used for each treatment. RNA-seq libraries were prepared using SMART-Seq HT Kit (Takara Bio) and Nextera XT DNA Library Preparation Kit (Illumina) and sequenced on an Illumina NovaSeq 6000 platform in the 100 bp paired-end mode. Base calling was performed using bcl2fastq conversion software ver.2.20 (Illumina). Sequenced reads were mapped to mouse reference genome sequences (mm10) using Hisat2 ver. 2.2.1, SAMtools ver. 1.3.1, and Stringtie ver. 2.1.7. Normalization was performed using EdgeR ver. 4.0.2 on R ver. 4.3.2. Differentially expressed genes (DEGs) between the groups were defined if they had a false discovery rate (FDR) of <0.05 and log_2_ (fold change) of less than −0.58 for downregulated genes or log_2_ (fold change) >0.58 for upregulated genes. Pathway analysis was performed with Ingenuity pathway analysis (IPA; Qiagen).

### Syt4 shRNA and cDNA expressing plasmids and adeno-associated virus preparation

Adeno-associated virus (AAVs) encoding U6-control shRNA-EF1α-EYFP were generated using the pAAV-shRNA-ctrl-EYFP plasmid, which was gifted by Hongjun Song (Addgene plasmid # 85741; http://n2t.net/addgene:85741; RRID: Addgene_85741; Yu et al., 2015). To generate shRNA constructs against mouse Syt4 mRNA (shSyt4), single DNA strands were annealed and ligated into the pAAV-shRNA-ctrl-EYFP plasmid, whose control shRNA sequences were removed by digestion with BamHI and XbaI to generate pAAV-shSyt4-EYFP plasmids. The same sequences used for siSyt4#1 and siSyt4#2 were used for shSyt4#1 and shSyt4#2, respectively. The knockdown efficiency of shSyt4 was validated by transfection of Neuro2a cells. To generate Syt4-T2A-EYFP expressing constructs, mouse Syt4 cDNA was amplified with RNA from C57BL/6J mouse brains, EYFP was amplified from pAAV-shRNA-ctrl plasmids, and the human synapsin 1 gene promoter (hSyn) was amplified from the pAAV-hSyn-DIO-hM4D(Gi)-mCherry plasmid (Addgene plasmid #44362; http://n2t.net/addgene:44362; RRID:Addgene_44362; Krashes et al., 2011) using PCR. Oligo DNA including T2A sequences was artificially synthesized (Fasmac). These four fragments were cloned into EcoRV-MluI site of pAAV-shRNA-ctrl-EYFP plasmids using In-Fusion HD Cloning Kit (Takara Bio) with appropriate order (hSyn-Syt4-T2A-EYFP). To generate the control vector (hSyn-T2A-EYFP), three fragments without Syt4 cDNA were cloned into EcoRV-MluI site of pAAV-shRNA-ctrl-EYFP plasmids.

To generate AAV9, AAVpro 293T cells (Takara Bio) were cotransfected with the three plasmids (pAAV-shRNA-ctrl, -shSyt4, hSyn-T2A-EYFP, or hSyn-Syt4-T2A-EYFP; pAAV2/9n; and pHelper) in a 1:1:2.5 weight ratio using Polyethylenimine Max (Polysciences). The pAAV2/9n plasmid was a gift from James M. Wilson (Addgene plasmid #112865; http://n2t.net/addgene:112865; RRID: Addgene_112865). Cells were collected 3 d later, and the AAVs were purified using an AAVpro Purification Kit (Takara Bio) according to the manufacturer's protocol. The AAVs were then aliquoted and stored at −80°C. Vector titers were determined as previously described (Aurnhammer et al., 2012). Briefly, the virus genome was purified by proteinase K treatment and ethanol precipitation and amplified with the KAPA SYBR Fast qPCR kit (KAPA Biosystems) and inverted terminal repeat primers. The titers were estimated to be between 4 and 7 × 10^13^ vg/ml.

### Spinal cord injury and AAV injection

Eight-week-old female mice were anesthetized using isoflurane and underwent laminectomy at the Th10 vertebral level. Dorsal hemisection was performed at Th10, with a lesion depth of 1 mm. After spinal cord transection, urinary bladders were manually expressed once daily until spontaneous voiding bladder contractions reappeared.

To visualize the projection of Syt4-silenced or Syt4-overexpressed corticospinal tract (CST), AAV-*Syt4* shRNA-EYFP, AAV-*Control* shRNA-EYFP, AAV-hSyn-Syt4-T2A-EYFP, or AAV-hSyn-T2A-EYFP was injected into the hindlimb area of the motor cortex (coordinates from the bregma: 0.5 mm posterior/0.5 mm lateral, 0.5 mm posterior/1.0 mm lateral, 1.0 mm posterior/0.5 mm lateral, and 1.0 mm posterior/1.0 mm lateral; 0.4 μl per site, all at a depth of 0.5–0.7 mm into the cortex) using a glass capillary attached to a microsyringe or a microinjector (BJ-120, BEX).

### Behavioral assessment

To assess hindlimb function, the mice were tested using the ladder walking test (Kuroda et al., 2017) every 7 d between Days 21 and 56 after spinal cord injury (SCI). A horizontal ladder (whole length of 1 m) with stainless steel rungs spaced 1–4 cm apart was used to evaluate hindlimb performance. Mice were habituated to the apparatus before surgery. The number of faulty placements in the injured hindpaw was counted when the mice walked through the 1 m stretch. Deep slips/misses, slight slips, and placement errors (correction, replacement, and correct placement) were considered faults (Metz and Whishaw, 2009). Control baseline scores were obtained immediately before injury. The spacing between the rungs was changed accordingly to prevent the animals from learning the rung locations. Behavioral testing was conducted between 1 P.M. and 6 P.M. The experiments were run in a blinded fashion.

### Western blotting

Mice injected with recombinant AAV vectors were deeply anesthetized and transcardially perfused with PBS. Their brains were harvested, and the sensory motor cortex with EYFP expression was excised under a fluorescent stereomicroscope. Tissues were lysed in lysis buffer containing 10 mM Tris-HCl, pH 7.4, 150 mM NaCl, 1% Triton X-100, and 1 mM ethylenediaminetetraacetic acid containing a protease inhibitor (Roche). The samples were resolved by sodium dodecyl sulfate-sulfate-polyacrylamide gel electrophoresis and transferred onto polyvinylidene difluoride membranes (Immobilon-P, Merck Millipore). The membranes were incubated with antibodies against rabbit anti-Syt4 (1:1,000, 105143, Synaptic Systems) or β-actin (1:5,000, #4970, Cell Signaling Technology). For detection, horseradish peroxidase-conjugated secondary antibodies (Cell Signaling Technology) and a chemiluminescent horseradish peroxidase substrate (WBKLS0500, Merck Millipore) were used. The signal was imaged using LAS-4000 (Fujifilm), and protein expression was quantified using ImageJ software. Values were normalized to the integrated density of β-actin bands.

### Immunohistochemistry

Cryosections were prepared from a fixed, sucrose-infiltrated frozen brain or the spinal cord. The mice were transcardially perfused with PBS and 4% paraformaldehyde in PBS. The isolated samples were post-fixed with 4% paraformaldehyde in PBS overnight at 4°C and transferred to 30% sucrose in PBS overnight. Tissues were embedded in optimal cutting temperature compound, frozen, sectioned at 25 μm (for the cervical spinal cord) and 20 μm (for other spinal cord sections) with a cryostat, and then mounted onto adhesive silane-coated glass slides (Matsunami Glass). For immunohistochemical analysis, the sections were permeabilized and blocked with PBS containing 0.2% Triton X-100 and 3% normal donkey serum for 1 h at 20–25°C. The sections were incubated with primary antibodies overnight at 4°C and then incubated with fluorescently labeled secondary antibodies for 1 h at 20–25°C. For Syt4 staining, sections were pretreated for antigen retrieval by boiling in 10 mM citric acid solution, pH 6.0, at 95°C for 10 min before permeabilization. The primary antibodies used were rabbit anti-Syt4 (1:1,000, 105143, Synaptic Systems), mouse anti-NeuN (1:1,000, MAB377, Merck Millipore), rabbit anti-Iba1 (1:1,000, 011-27991, Wako), goat anti-SOX9 (1:200, AF3075, R&D Systems), mouse anti-Olig2 (1:1,000, MABN50, Merck Millipore), rabbit anti-GFP (1:4,000, ab6556, Abcam), rat anti-GFP (1:1,000, 04404-26, Nacalai Tesque), rabbit anti-PKCγ (1:1,000, 59090, Cell Signaling Technology), and rat anti-GFAP (1:1,000, 13-0300, Invitrogen) antibodies. Alexa Fluor 488-conjugated donkey antibody against rabbit IgG, Alexa Fluor 568-conjugated donkey antibody against mouse IgG, Alexa Fluor 647-conjugated donkey antibody against goat IgG, and Alexa Fluor 594-conjugated donkey antibody against rat IgG (Thermo Fisher Scientific) were used as secondary antibodies. Nuclear staining was performed using 1 μg/ml DAPI. Images were acquired using a confocal laser scanning microscope (FluoView FV3000, Olympus).

To assess the localization of Syt4 in the motor cortex, brain sections ranging from −0.5 to −1.0 mm from the bregma were obtained from 8-week-old female C57BL/6J mice. For quantification of glial scar formation after SCI, the percentage of GFAP-positive and Iba1-positive regions were quantified in 1.5 × 1.5 mm^2^ squares centered on the lesion site.

### Quantitation of tracing fibers

To quantify CST sprouting in the cervical spinal cord, the number of EYFP-labeled CST collaterals crossing the line positioned in the gray matter was counted using a horizontal section prepared from segments C4 to C7. To exclude the difference in tracing efficiency, the number of CST collaterals per section was normalized by the total number of labeled CST axons; three sections spaced 100 µm apart were examined, and the average number of CST collaterals per section was divided by the number of labeled CST axons at the medulla. The statistical outliers in technical replicates were identified by Grubb's test and removed from statistical analysis.

To assess CST projection around the lesion, the intensity of the EYFP signal was measured every 100 μm from the lesion site in three serial 20-µm-thick sections from the thoracic spinal cord. The values were normalized with the intensity of the images at 1.0 mm rostral to the injury site.

### Statistical analysis

Data are presented as mean ± standard error of the mean. Significant differences between groups were determined by an unpaired Student's *t* test or repeated-measures analysis of variance (ANOVA), followed by post hoc comparisons with the Tukey–Kramer test. *p* < 0.05 was considered statistically significant.

## Results

### Syt4 is involved in the neurite elongation of cortical neurons in vitro

To identify novel candidate genes involved in the formation of compensatory neural networks in neurological disorders, several datasets were combined using public databases (Fig. 1*A*). We first performed phenotype-based screening using Online Mendelian Inheritance in Man (OMIM; https://omim.org/) and selected 1,000 genes that were output as a result of inputting “motor dysfunction” to identify genes related to functional recovery after SCI. We then extracted the top 60 genes that were mainly expressed in the brain, using RNA expression profiles from the Human Protein Atlas (https://www.proteinatlas.org/). Among them, we additionally selected the top 20 genes that showed abundant expression in the neuron compared with the other cell types using Brain RNA-seq (https://www.brainrnaseq.org/; Fig. 1*A*). For functional recovery after SCI, axon regrowth from the remaining neuronal network is required to form compensatory networks (Filli and Schwab, 2015). Thus, we identified genes associated with neurite elongation by siRNA screening in primary mouse cortical neurons using an siRNA library consisting of siRNA pools that included four distinct siRNAs targeting different gene regions (Table 1). To mimic in vivo axonal injury, siRNA-transfected neurons were trypsinized and repeated so that axons elongated prior to gene silencing were injured and removed from the culture. Neurite length was measured after immunocytochemistry for Tuj1, and the neurons transfected with *Syt4* siRNA pool showed highest inhibition effect on neurite outgrowth (Table 1). Furthermore, we also detected that *Syt4* siRNA pool transfection extensively prevented the neurite elongation in Ctip2^+^ layer V neurons compared with that in the control (*Control* siRNA pool; Fig. 1*B*,*C*). Notably, siRNAs with two independent target sequences for Syt4 (*Syt4* siRNA#1 and *Syt4* siRNA#2) inhibited neurite elongation (Fig. 1*B*,*C*), confirming the on-target effect of the siRNA experiments. We confirmed that *Syt4* expression was significantly reduced by transfection with *Syt4* siRNAs (Fig. 1*D*), and the number of neurons was not changed by *Syt4* siRNAs (Fig. 1*E*).

To investigate the mechanism of neurite elongation by *Syt4*, we performed RNA sequencing (RNA-seq) of primary cortical neurons transfected with *Control* or *Syt4* siRNA pool. The volcano plot showed the fold change and expression level of each gene after *Syt4* siRNA transfection; 119 upregulated and 474 downregulated genes were detected (Fig. 1*F*). Gene ontology analysis indicated that these DEGs were involved in “neuron projection development (GO: 0031175)” and the “positive regulation of neuron projection development (GO: 0010976)” (Fig. 1*G*,*H*). A functional analysis mapping DEGs to signaling pathways identified potential signals whose reduction in activity with Syt4 silencing might cause inhibition of neurite elongation (Fig. 1*I*), including a known Sy4 downstream signaling “p38 MAPK signaling,” that has been reported to be involved in neurite outgrowth (Kase et al., 2022; Huang et al., 2024).

### Syt4 is expressed in neurons of the motor cortex

**Figure 2.**
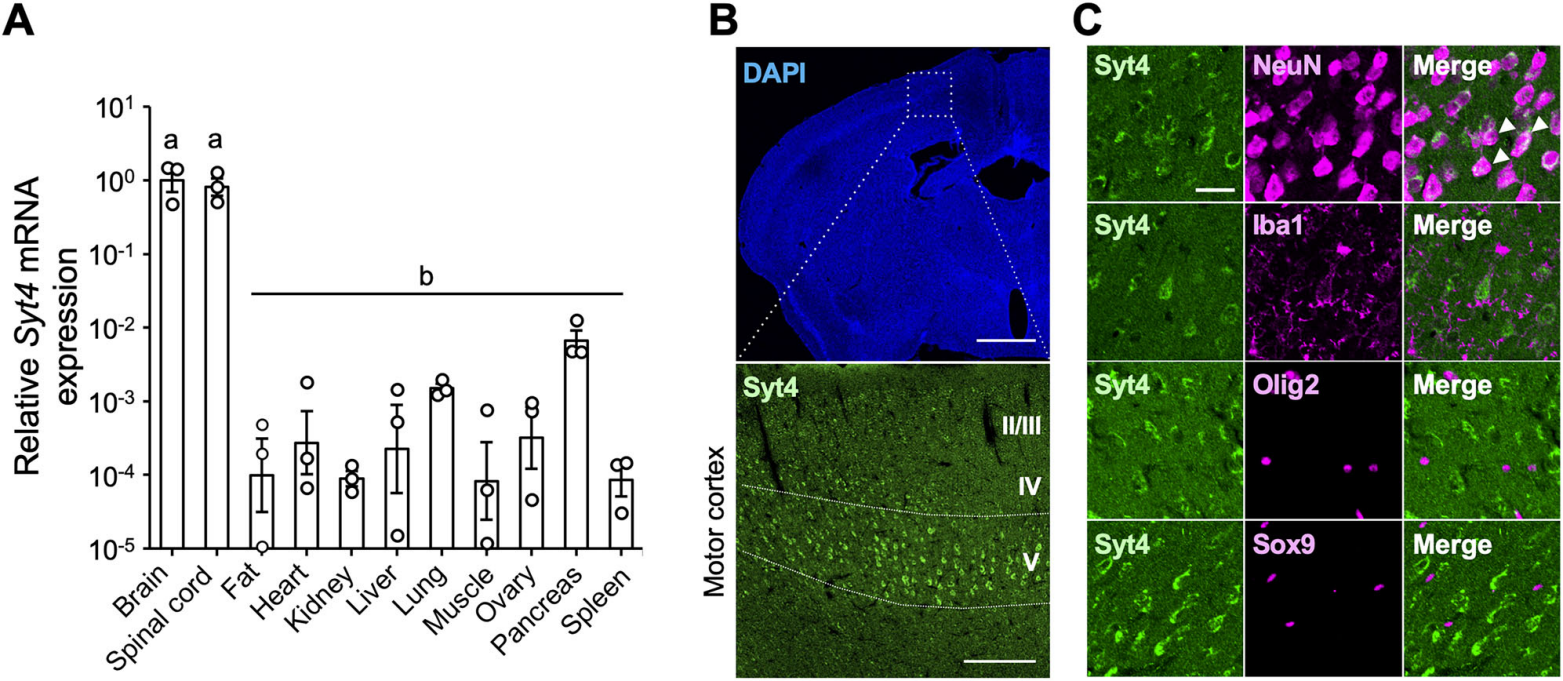
Syt4 is expressed in neurons of the primary motor cortex. ***A***, Relative *Syt4* mRNA expression in individual organs of intact mice (*n* = 3 each). Data are presented as mean ± standard error of the mean. Bars with different letters are significantly different (*p* < 0.05, one-way ANOVA with Tukey's post hoc test). ***B***, Low magnification view of brain section labeled with DAPI (top image) and immunohistochemistry for Syt4 in the motor cortex indicated with white rectangle in the top image. Scale bar: top, 1 mm; bottom, 100 μm. ***C***, Representative images show dual immunostaining for Syt4 (green) and NeuN, Iba1, Olig2, or Sox9 (magenta) in the motor cortex. Scale bar, 25 μm.

To validate the tissue specificity of *Syt4*, we investigated *Syt4* expression in various organs of adult mice. Quantitative RT-PCR analysis revealed that *Syt4* mRNA expression in the brain and spinal cord was much higher than in other organs, suggesting that *Syt4* is mainly expressed in the CNS (Fig. 2*A*). Immunohistochemistry showed that Syt4 was expressed in the motor cortex, especially in layer V, the origin of the CST (Fig. 2*B*). We also performed coimmunostaining for Syt4 with NeuN (neurons), Iba1 (microglia), Olig2 (oligodendrocyte-lineage cells), and Sox9 (astrocytes) to determine the cell type expressing Syt4. We demonstrated that the Syt4 signal was colocalized with 76.0 ± 5.6% of NeuN^+^ cells but not with Iba1, Olig2, or Sox9 (Fig. 2*C*).

### Syt4 contributes to functional recovery and collateral sprouting after SCI

**Figure 3.**
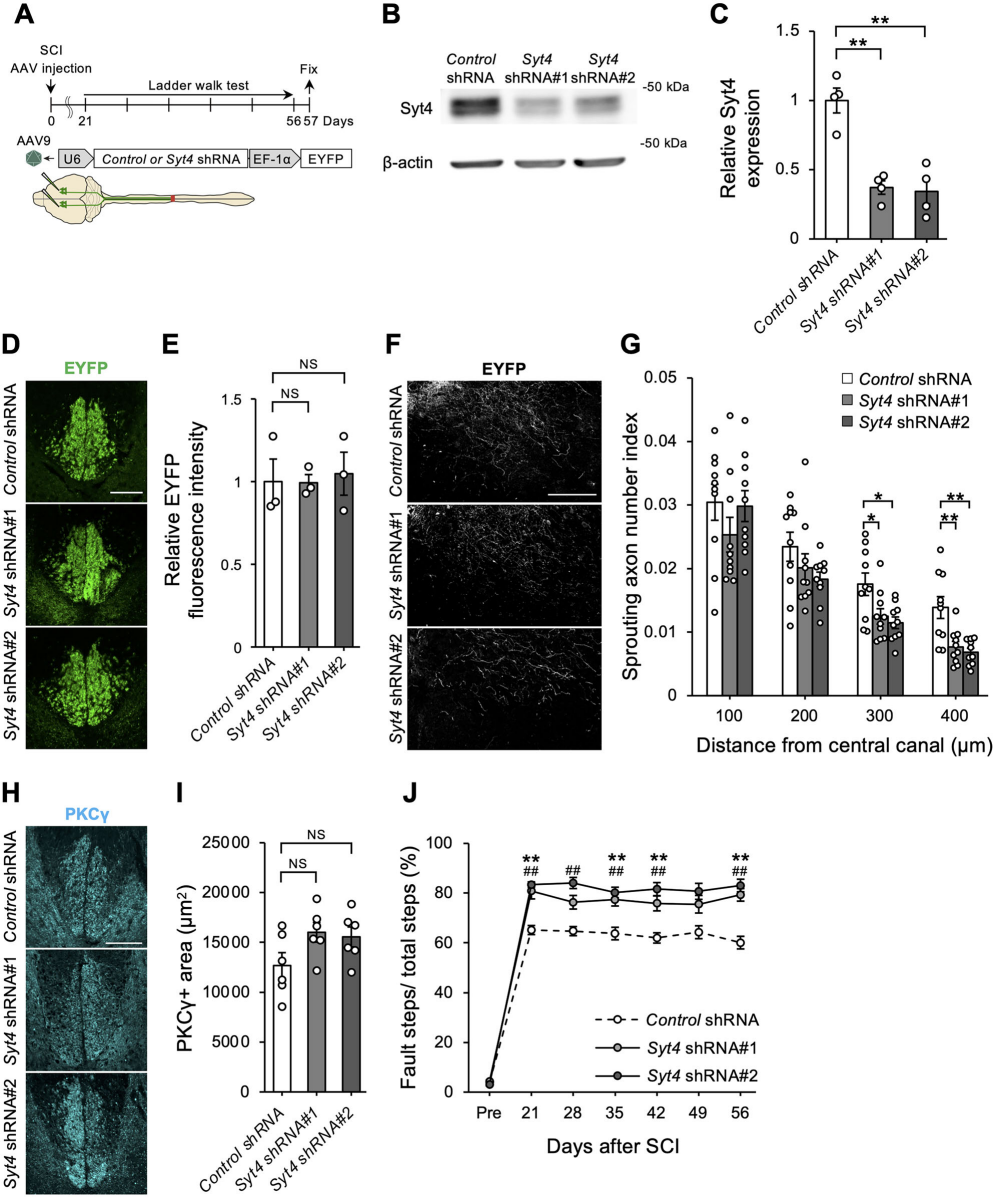
Endogenous Syt4 contributes to spontaneous functional recovery and collateral sprouting after SCI. ***A***, Schematic drawing indicating the timeline and AAV injection procedure for in vivo experiments. ***B*,** Western blots showing the expression level of Syt4 and β-actin in the motor cortex after 14 d of AAV infection. ***C***, Graph showing the relative expression level of Syt4 assessed by western blots. Data is normalized to the intensity of β-actin (*n *= 4 for each, **p < 0.01). ***D***, Representative images show EYFP-labeled CST in the dorsal columns of cervical cord of mice after 14 d of AAV infection. Scale bar, 100 μm. ***E***, Quantitative analysis of the relative EYFP fluorescence intensity assessed from ***D*** (*n *= 3 for each; NS, not significant). ***F***, Representative images show EYFP-labeled CST axons in the cervical cord at Day 57 after SCI. Scale bar, 200 μm. ***G***, Quantification of sprouting fiber index in the indicated distance from the central canal. The number of sprouting fibers was normalized to that of main CST fibers (*n *= 10 for each, *p < 0.05, **p < 0.01). ***H***, Representative images show immunohistochemistry for PKCγ in the cervical cord of mice at Day 57 after injury. Scale bar, 100 μm. ***I***, Quantitative analysis of PKCγ^+^ area (*n *= 6 for each; NS, not significant). ***J***, Graph showing the percentage of faults in ladder walk test after SCI (*Control* shRNA: *n *= 14, *Syt4* shRNA#1: *n *= 12, and *Syt4* shRNA#2: *n *= 12, **p < 0.01, *Control* shRNA vs *Syt4* shRNA#1, ^##^*p *< 0.01, *Control* shRNA vs *Syt4* shRNA#2). Data are presented as mean ± standard error of the mean. *p* values were determined by one-way ANOVA with Tukey's post hoc test (***C***, ***E***, ***I***) or two-way ANOVA with Bonferroni's post hoc test (***G***, ***J***).

To assess the in vivo effect of *Syt4*, we used an SCI mouse model with dorsal hemisection of the thoracic spinal cord (Fig. 3*A*). In this model, SCI disrupts the axonal network of the CST, a descending motor tract that causes severe neuronal dysfunction. However, CST axons spontaneously form sprouting fibers, which reorganize the compensatory neural network with connections to propriospinal neurons, resulting in spontaneous functional recovery (Bareyre et al., 2004). To examine whether *Syt4* mediates the formation of a compensatory neural network in the CST after SCI, we injected an adeno-associated virus-9 (AAV9) encoding *Control* shRNA, *Syt4* shRNA#1, or shRNA#2 with EYFP into the motor area of the cerebral cortex to reduce Syt4 protein expression (Fig. 3*B*,*C*). Silencing *Syt4* expression in CST of naive mice did not change their projections in dorsal column of cervical spinal cord (Fig. 3*D*,*E*), suggesting that Syt4 is dispensable for maintenance of axons in adult CNS. Subsequently, we conducted SCI simultaneously with injection of Syt4-silencing vectors and counted the number of EYFP-labeled sprouting fibers in the cervical spinal cord, which contribute to functional recovery after SCI (Haenzi et al., 2016; Sun et al., 2020). Quantitative analysis revealed that the number of sprouting fibers was decreased by both *Syt4* shRNA#1 and *Syt4* shRNA#2 treatment compared with the *Control* shRNA (Fig. 3*F*,*G*), indicating that endogenous Syt4 plays a role in sustaining the spontaneous formation of neuronal networks after injury. We confirmed that there was no significant difference in the PKCγ^+^ main CST area in the dorsal column of the spinal cord between the groups (Fig. 3*H*,I). SCI in the thoracic spinal cord causes hindlimb dysfunction with spontaneous recovery, depending on the reconstruction of the neuronal network (Ghosh et al., 2010). Thus, we then investigated whether Syt4-dependent neuronal network reconstruction contributed to functional recovery after injury. Functional analysis using a ladder walk (Nakanishi et al., 2019; Balçıkanlı et al., 2022) test revealed that mice treated with *Syt4* shRNA#1 and *Syt4* shRNA#2 showed higher fault steps than control animals even several weeks after injury (Fig. 3*J*), suggesting that silencing *Syt4* prevents functional recovery after SCI.

**Figure 4.**
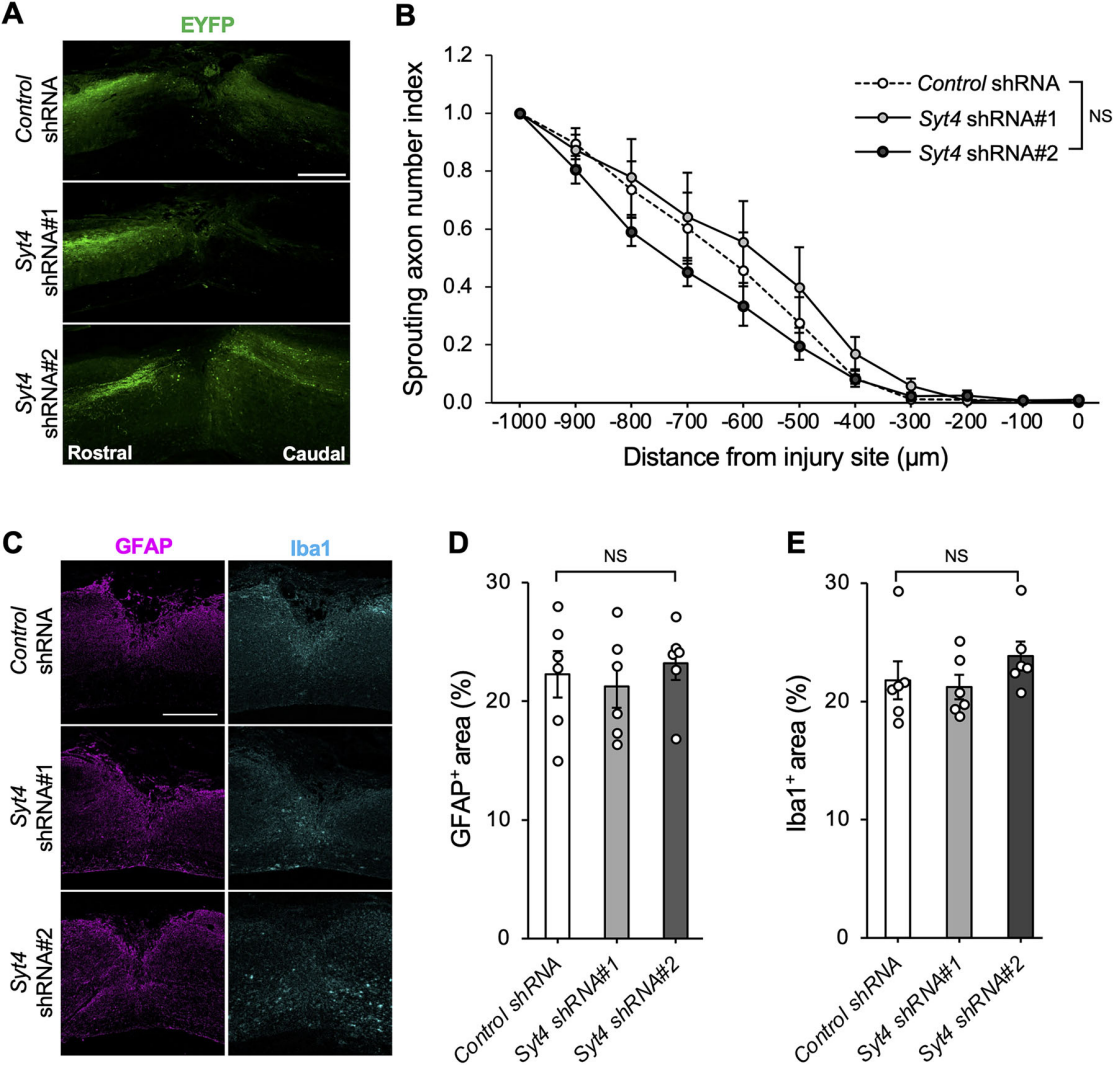
Syt4 knockdown does not affect histological feature around the lesion. ***A***, Representative images show EYFP-labeled CST axons around the lesion site at Day 57 after SCI. Scale bar, 500 μm. ***B***, Quantification of EYFP^+^ axon intensity at indicated distance from lesion site. The percentage of fluorescence intensity was calculated based on 1 mm rostral to the injury (*Control* shRNA: *n *= 6, *Syt4* shRNA#1: *n *= 5, and *Syt4* hRNA#2: *n *= 4 for each). ***C***, Representative images show the immunohistochemistry for GFAP (magenta) and Iba1 (cyan) in the lesion site at Day 57 after injury. Scale bar, 500 μm. ***D***, ***E***, Quantification of GFAP^+^ area (***D***) and Iba1^+^ area (***E***). The area index was calculated as the percentage of stained area per unit area (*n *= 6 for each). Data are presented as mean ± standard error of the mean. *p* values were determined by two-way (***B***) or one-way (***D***, ***E***) ANOVA; NS, not significant.

Next, we investigated whether Syt4-mediated functional recovery depended on other histological modifications. For the CST projection around the lesion site, we measured the intensity of the EYFP-labeled axons at the rostral lesion in the thoracic spinal cord at the end of the behavioral observation. EYFP fluorescence intensity did not differ between the *Control* shRNA and *Syt4* shRNA#1-or *Syt4* shRNA#2-treated mice (Fig. 4*A*,B). We also determined the effect of Syt4 knockdown on glial scar formation, which inhibits the regrowth of damaged axons (Tran et al., 2018); however, we did not find a significant difference in the area of GFAP^+^ astrocytic gliosis between groups (Fig. 4*C*,*D*). We also detected no difference in the accumulation of Iba1^+^ microglia between groups (Fig. 4*C*,*E*).

### Syt4 is sufficient to promote functional recovery and collateral sprouting after SCI

**Figure 5.**
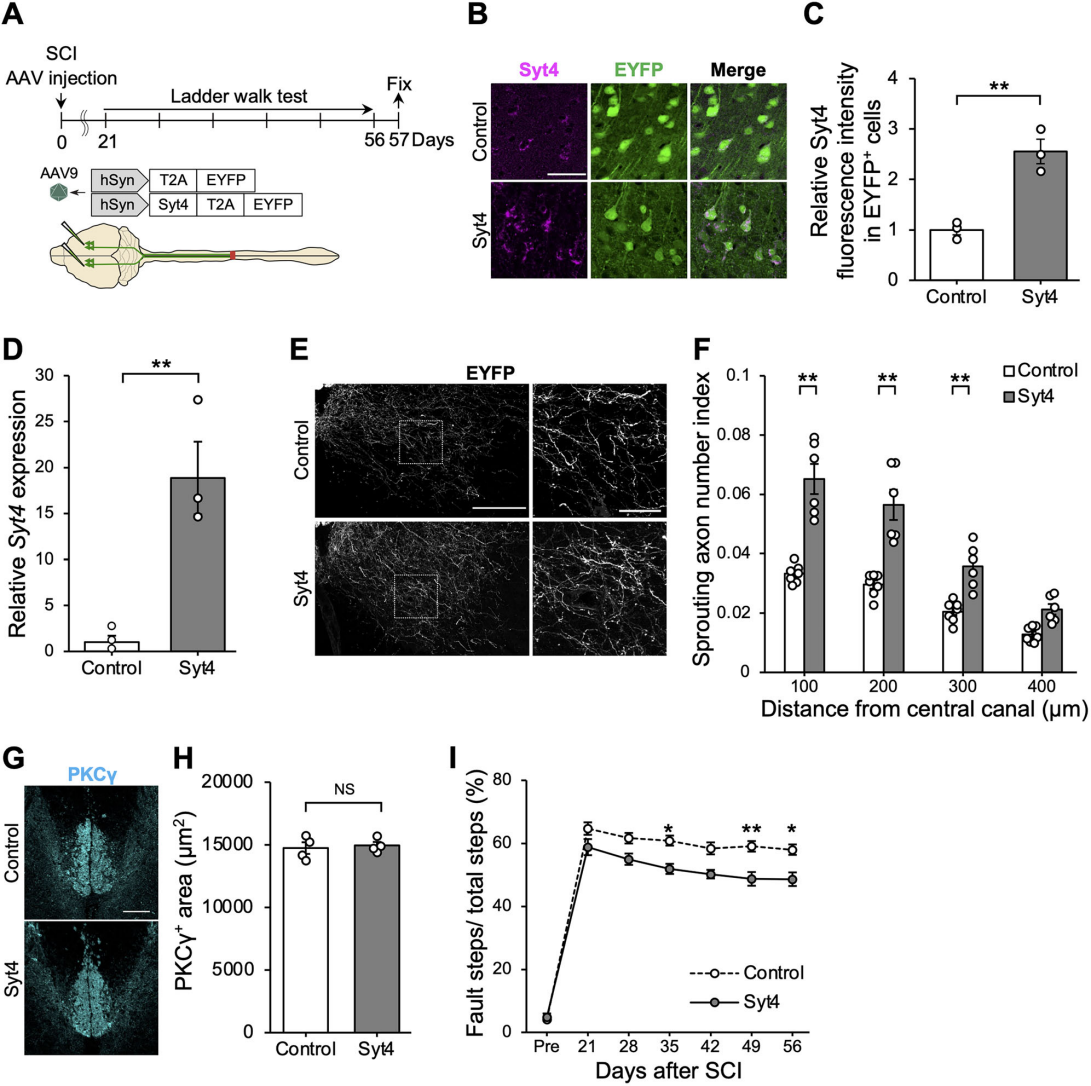
Syt4 overexpression is sufficient to promote functional recovery and collateral sprouting after SCI. ***A***, Schematic drawing indicating the timeline and AAV injection procedure for in vivo Syt4-overexpressing experiments. ***B***, Representative images of immunohistochemical analysis showing the expression level of Syt4 in the motor cortex after 14 d of AAV infection. Scale bar, 50 μm. ***C***, Graph showing the relative expression level of Syt4 in EYFP^+^ transduced neurons assessed by immunofluorescence (*n *= 3 for each; **p < 0.01). ***D***, Graph showing the relative expression level of *Syt4* mRNA in the motor cortex after 14 d of AAV infection (*n *= 3 for each; **p < 0.01). ***E***, Representative images show EYFP-labeled CST axons in the cervical cord at Day 57 after SCI. High magnification images of the boxed areas in the left panels are shown in the right panels. Scale bars: 200 μm for the left panels and 50 μm for the right panels. ***F***, Quantification of sprouting fiber index in the indicated distance from the central canal. The number of sprouting fibers was normalized to that of main CST fibers (Control: *n *= 7, Syt4: *n* = 6, **p < 0.01). ***G***, Representative images show immunohistochemistry for PKCγ in the cervical cord of mice at Day 57 after injury. Scale bar, 100 μm. ***H***, Quantitative analysis of PKCγ^+^ area (*n *= 4 for each; NS, not significant). ***I***, Graph showing the percentage of faults in ladder walk test after SCI (Control: *n *= 7; Syt4: *n* = 6; *p < 0.05, **p < 0.01). Data are presented as mean ± standard error of the mean. *p* values were determined by Student's *t* test (***C***, ***D***, ***H***) or two-way ANOVA with Bonferroni’s post hoc test (***F***, ***I***).

Finally, we investigated whether the activation of Syt4 function facilitates axon sprouting and functional recover after SCI. We injected AAV9 encoding mouse Syt4 cDNA with coexpression of EYFP via T2A linker into the motor area of the cerebral cortex to overexpress Syt4 (Fig. 5*A*). The transgenes were designed for expression under the control of the hSyn promoter, which confers neuron-specific expression (Kügler et al., 2003; Fig. *5B*,*D*). We simultaneously conducted SCI and counted the number of EYFP-labeled sprouting fibers in the cervical spinal cord. Quantitative analysis revealed that the number of sprouting fibers was increased upon the injection of Syt4-overexpressing vectors compared with the control vectors (Fig. 5*E*,*F*), without changes in PKCγ^+^ main CST area in the dorsal column of the spinal cord (Fig. 5*G*,*H*). The percentages of faults in the ladder walk test were remarkably smaller in the Syt4-overexpressed animals compared with the controls (Fig. 5*I*), indicating that overexpression of Syt4 promotes the reconstruction of neuronal networks and contributes to functional recovery after injury.

## Discussion

In summary, we identified Syt4 as a novel regulator of axon regrowth following CNS injury. We applied loss-of-function genomic screening alongside Web resource-based phenotypic screening to find that Syt4 knockdown inhibited neurite outgrowth in cortical neurons. We also observed that Syt4 knockdown in the CST inhibited compensatory neural network formation and functional recovery after SCI.

Syt4 is a member of the synaptotagmin family, which plays a key role as a calcium sensor in calcium-dependent exocytosis and endocytosis (Chapman, 2002; Mori and Fukuda, 2011). Although Syt4 expression has been detected in the Golgi apparatus and tips of growing neurites in developing hippocampal neurons (Ibata et al., 2000), its role in neurite outgrowth has not been elucidated. In addition to regulating neurite outgrowth by synaptotagmins, Syt17 (an isoform of the Syt family) interacts with Golgi proteins and promotes neurite outgrowth by regulating early secretory trafficking (Ruhl et al., 2019). Syt4 has also been detected in the Golgi apparatus and dense core vesicles (Ibata et al., 2000; Bharat et al., 2017), suggesting that Syt4 promotes neurite outgrowth by regulating the transport of key molecules and organelles during this process. However, considering that Syt family members have been reported to function in neurons in a noncomplementary manner (Adolfsen et al., 2004), further precise investigations are needed to elucidate how Syt4 functions in compensatory neuronal network formation. We found that Syt4 knockdown altered the expression of genes involved in signaling pathways associated with neurite outgrowth. This observation suggests that Syt4 also mediates intracellular signaling, resulting in changes in gene expression. If so, how Syt4 changes gene expression in neurons remains unclear. Reportedly, Syt4 promotes melanogenesis by regulating intercellular calcium influx via the transient receptor potential melastatin 1 (TRPM1) channel; it also regulates expression or phosphorylation of calcium signaling molecules, such as calcium/calmodulin-dependent protein kinase IV (CAMK4), extracellular regulated MAP kinase (ERK), and p38 (Jia et al., 2020; Huang et al., 2024). Therefore, Syt4 may mediate neurite outgrowth via multiple signal transduction pathways induced by calcium influx. Of note, our RNA-seq analysis followed by a functional analysis proposed reduction in p38 MAPK signaling activity with Syt4 silencing. As p38 activity has been reported to be crucial in neurite elongation (Kase et al., 2022), it is a potent candidate for mediator of Syt4-dependent neurite outgrowth.

Our in vitro experiments showed that *Syt4* in cortical neurons inhibited neurite outgrowth, suggesting that Syt4-expressing cells autonomously regulate axonal regrowth after SCI. Although we detected Syt4 protein expression predominantly in neurons of the motor cortex, *Syt4* transcript expression is not limited to the neurons in the CNS but is rather also expressed in other types of cells, such as astrocytes, albeit at lower levels (Mittelsteadt et al., 2009; Y. Zhang et al., 2014). Therefore, axonal regrowth inhibition by Syt4 knockdown in the neocortex is possibly regulated by a noncell autonomous mechanism. Indeed, Syt4 has been reported to regulate glutamate release in astrocytes (Q. Zhang et al., 2004). Cortical astrocytes have been known to play a role in compensatory neuroplasticity after CNS damage by secreting factors that regulate synaptogenesis, neurogenesis, and axonal remodeling (Zhou et al., 2020; Heras-Romero et al., 2022). Although our in vitro experiments showed evidence that Syt4 acts on neurons, further studies may elucidate the involvement of other cell types in Syt4-mediated compensatory neuronal network formation. According to the link between Syt4 and functional recovery, *Syt4* null mutant mice exhibit deficiencies in learning, memory, and motor coordination (Ferguson et al., 2000). At the cellular level, the role of Syt4 in synaptogenesis and synaptic function is well characterized (Yoshihara et al., 2005; Dean et al., 2009; Bharat et al., 2017). However, to the best of our knowledge, there are no reports on the direct link between axonal growth and Syt4.

An intriguing question is how Syt4 contributes to compensatory neuronal networks and functional recovery after SCI. We observed that silencing Syt4 prevents axon sprouting in the cervical spinal cord, however, change their projection around the lesion in the spinal cord. It is known that there are many factors that inhibit axon regeneration around the lesion site depending on the formation of scar in the response to injury (Ahuja et al., 2017). Therefore, the variation observed in the reconstruction of the CST projection within different regions may arise from dissimilar microenvironments surrounding the CST axons. As per the axon sprouting in the cervical spinal cord, we found that silencing *Syt4* inhibited sprouting fiber elongation toward the distal area, but not the proximal area, from main CST. The process of axon sprouting after injury starts with the formation of axon arborization to make collaterals. After which, collaterals extend toward target neurons situated in distal areas, traversing from the central canal, in order to establish compensatory neural circuits (Bareyre et al., 2004; Collyer et al., 2014). Hence, the finding that the reduction of Syt4 expression significantly decreased the number of sprouting fibers solely in the distal area suggests that Syt4 primarily contributes to the elongation or stabilization of sprouting fibers, while having no impact on the formation of arborization from the remaining main CST axons. This hypothesis is supported by studies reporting that elongation of sprouting fibers toward the distal area could be modulated by molecules promoting neurite outgrowth (Lang et al., 2013) or via the stabilization of elongated neurites (Bareyre et al., 2004). On the other hand, we found that overexpression of Syt4 increased sprouting fibers also in the proximal part, suggesting that ectopic Syt4 expression has potential to promote axon branching as well as its elongation. Besides the intercellular mechanisms, we found that silencing *Syt4* inhibited neurite outgrowth in cultured neurons before synapse formation, suggesting that Syt4 regulates axonal reconstruction via direct control of axon elongation. In contrast, synaptic remodeling of corticospinal neurons occurs after SCI (Kim et al., 2006; Ghosh et al., 2009, 2010). Thus, Syt4-mediated regulation of synapse reorganization may contribute to the formation of a compensatory neuronal network (Kim et al., 2008). Further precise investigation is needed to elucidate the mechanism by which Syt4 regulates reconstruction of neural circuit.

Axon degeneration is a hallmark of other CNS diseases, such as Parkinson's disease (Burke and O’Malley, 2013), Alzheimer's disease (Kanaan et al., 2013), and ischemic stroke (Hinman, 2014). Syt4 has been implicated in these diseases, and genome-wide association studies have found single nucleotide polymorphisms associated with Parkinson's disease (Chang et al., 2017) and Alzheimer's disease (Jansen et al., 2019); altered Syt4 expression has also been observed in an experimental model of ischemia (Yokota et al., 2001). Further evaluation of the multiple roles of Syt4 in CNS regeneration may contribute to unveiling novel molecular targets for treating CNS pathologies with axon degeneration.
